# Notes and News

**Published:** 1892-02-20

**Authors:** 


### Notes and News.

Collected and Communicated.

The Master of St. Katharine's, President of Queen Victoria's Jubilee Institute for Nurses, will discourse on the "Queen Victoria's Jubilee Institute for Nurses: the Object and the Work," on Wednesday the 24th instant, at Westminster Hospital, at 8 p.m. Mr. T. Bryant, President of the Royal College of Surgeons, in the chair.

The eighth annual report of the Princess Alice Memorial Hospital, Eastbourne, is satisfactory. During the year the Alexandra wing has been completed and opened. The expenditure amounted to £1,327, against an income of £1,335. 226 in-patients were admitted to treatment. We are sorry to notice the omission of any recognition of the valuable services rendered to the Institution by Miss Hamilton, the Matron.

Forty-two in-patients and 161 out-patients were treated at the Dunster and Minehead Village Hospital during 1891, at a total cost of £268. We are glad to notice that the medical officers while strongly expressing an opinion that the in-patient department is essentially part of cottage hospitals in rural districts, declare that it is doubtful if the out-patient department is a necessity at all, and that if retrenchment be called for they should have no hesitation in closing it entirely.

The Hospital Sunday Fund is growing at Hull. This year £800 has been collected by 105 congregations (an increase of fifteen for the year) which contributed collectively £173 more than in 1890. Holy Trinity Church contributed the largest collection, viz., £66 3s. Of the total collection the Church of England contributed £398, Wesleyans £165, the Primitive Methodists £51, the Congregationalists £47 Roman Catholics £45, the Jews £26, the Presbyterians £17, the Unitarians £10, Danish and German Lutheran Churches £10, Friends £9, Baptists and various other congregations £21. Of the whole sum the Royal Infirmary received £597 as its share. We congratulate the committee of the fund on the progress made, and hope it may continue. The Infirmary receives large sums from other churches near Hull, their contributions amounting in 1890 to no less than £924. It might be well, therefore, for the Hospital Sunday Fund authorities to adopt the London plan of requesting the participating institutions to state in their accounts the amount received each year for periodical collections apart from Hospital Sunday.

The movement for the establishment of a general hospital at Kettering is progressing satisfactorily. Already upwards of £3,000 out of the £4,500 required before a commencement is made, has been promised. The Duke of Buccleuch has given two acres of land, forming an excellent site for the new hospital, and it is intended to affiliate with it a dispensary and out-patient department, and also a home and training school for nurses. Mr. J. T. Iliffe is the Hon. Secretary, and if anyone interested in Kettering reads this paragraph, we hope they will promptly send him a cheque, as this hospital is greatly needed.

Dr. William Ogle has again protested against the appointment of a general practitioner as physician to the Derbyshire Royal Infirmary. He declares that the town and neighbourhood of Derby sadly need additional consulting powers, as those already available "have been for many years notoriously and discreditably feeble." He urges that "the hospital exists primarily for the care and cure of patients, and not for the purposes of a few individuals who have combined together for the express purpose of preventing anything being done which they do not favour, or which they are pleased to think opposed to their own personal interest." We regret the quarrel, but are inclined to agree that an important city like Derby should, at any rate, have the advantage of one practising consulting physician, though it ought to be able to support, and would no doubt support, if it knew its own best interests, three or four pure consultants at least.

The authorities of the Devonshire Hospital and Buxton Bath Charity have issued their report for 1891 with commendable expedition. 2,351 in-patients, and only 182 out-patients were received. The average cost per week of each patient has been 15s. instead of 15s. 4d. last year, and £396 has been expended in the maintenance of the baths connected with the hospital. The income amounted to £6,300, of which only £118 was received from congregational collections. Legacies amounted to £900; and £2,826 was received from annual subscribers, in addition to £1,195 from casual subscriptions, though we do not understand the difference between these two sources of income. Buxton contains an unusual number of hotels, and it is satisfactory to note that nearly £200 was received from this source. Buildings, including the baths, have cost nearly £52,000, and the invested property amounts to £10,636. As showing the special treatment, it may be mentioned that eight out of every nine patients used the baths, and the remaining ninth were patients suffering from accidents or advanced pulmonary disease.

Mr. F. Richardson Cross is to be congratulated upon the steady progress made by the Bristol Eye Hospital, which he has done so much to develop. Two hundred and forty-two in-patients were admitted last year, the daily average number of beds occupied being sixteen. It has been found that this number is inadequate to meet the demands, and that in order at times to treat successfully and with economy some of the more serious cases, and to allow a few days' rest in the hospital in cases of urgency during the most critical period, of their maladies, another ten beds are required. It has therefore been determined to enlarge the hospital at a cost of £2,000, of which sum about one-half has already been raised. The Bristol Eye Hospital serves a large district in the west of England, and it should be as easy to raise so small a sum for this object as to write a letter or to eat one's dinner. The Bristol Eye Hospital was the third hospital established in this country for diseases of the eye, Moorfields being opened in 1804, and the Eye Hospital, at Exeter, a year or two later. We hope Mr. Richardson Cross will get all the money he requires by Easter at the latest.

The movement to commemorate the memory of the late Mr. John Morgan, an influential general practitioner practising in Paddington, of which Miss Ethel M. Trowe is the able Hon. Secretary, has resulted in a sum of £1,136. The Committee charged with the movement have sent a cheque for this amount to Sir James Paget, the President of the British Medical Benevolent Fund, with a request that the Council will invest it to form a "John Morgan Annuity," to be given if possible to a widow with children, who shall enjoy the annuity till such time as the Council may consider her children capable of supporting their mother. The work of the general practitioner is most arduous, and the spontaneous and prompt manner in which the patients of the late Mr. Morgan have raised this affectionate tribute to his memory is gratifying, as it is worthy of imitation by other communities all over the country.

The State Children's Relief Department of the New South Wales Government is charged with the supervision and management of the boarding-out system in this colony. The advantages of family life for the orphans and destitute children of the colony, as opposed to the unnatural method of training them in large institutions, is proved by the successful results realised in New South Wales. The same system prevails in Victoria and South Australia, and it has been partly introduced with great success into Queensland and New Zealand. There is at present a want of uniformity of control in New South Wales owing to the existence of three distinct departments, the boarded-out children being under the direction of the Colonial Secretary, the Girls' Reformatory under the Minister of Justice, and the Industrial Schools (which are practically penal establishments) under the Minister of Public Instruction. Victoria and South Australia have had the wisdom to place their children under the control of a single organisation, and we shall hope to see this plan soon adopted in New South Wales. Mr. Waugh will be glad to know that a Society for the Prevention of Cruelty to Children was recently established in Sydney, but the work is very considerably hampered by the absence of a law regulating such matters. The report of the President of the State Children's Relief Department states that the "home life of the children at present is a blot upon our civilisation, that they are the victims of unkindness and neglect in the worst form, a state of affairs which will never be remedied until Parliament deals with the question effectually." Up to April 5th, 1891, 3,910 children have been removed from public institutions and boarded-out in New South Wales, the number of children at present under care being 2,369; 1,373 of the children are at present supported by the Government, 158 have been adopted without payment, and 838 have been apprenticed to trades. The Treasury contributed £27,135 towards the maintenance of the system, of which £26,330 was expended, the gross average cost per child being £14 6s. 10d. The moral results of the system are stated to be surprisingly good, but there is still much need of help from lady visitors and those who exercise a voluntary oversight and inspect periodically the homes where the children are placed.

